# Gut microbiome and fecal metabolic alteration in systemic lupus erythematosus patients with depression

**DOI:** 10.3389/fcimb.2022.1040211

**Published:** 2022-11-25

**Authors:** Han Yao, Hao Yang, Yueying Wang, Qian Xing, Lin Yan, Yaru Chai

**Affiliations:** ^1^ Department of Immunology and Rheumatology, Qingdao Municipal Hospital Affiliated to Qingdao University, Qingdao, Shandong, China; ^2^ School of Clinical Medicine, Weifang Medical University, Weifang, Shandong, China; ^3^ School of Clinical Medicine, Graduate School of Dalian Medical University, Dalian, Liaoning, China

**Keywords:** systemic lupus erythematosus, depression, gut microbiome, fecal metabolites, cytoinflammatory factor, BDNF

## Abstract

**Background:**

Mental health disorders in systemic lupus erythematosus (SLE) are gradually getting recognized; however, less is known regarding the actual structure and compositional alterations in gut microbiome and metabolism and the mechanisms of how they affect depression development in SLE patients.

**Methods:**

Twenty-one SLE patients with depression (SLE-d), 17 SLE patients without depression (SLE-nd), and 32 healthy controls (HC) were included in this study. Fecal samples were collected for 16S rRNA gene sequencing and ultra-high-performance liquid chromatography-quadrupole time-of-flight mass spectrometry (UHPLC-QTOF-MS) based metabolomics.

**Results:**

The structure of gut microbiome in the SLE-d group changed compared with that in the other two groups. The microbiome composition of SLE-d group showed decreased species richness indices, characterized by low ACE and Chao1 indices, a decrease in the ratio of phylum Firmicutes to Bacteroidetes, genus *Faecalibacterium* and *Roseburia*. A downregulation of the metabolite fexofenadine involved in bile secretion was positively correlated with the genus *Faecalibacterium*, *Subdoligranulum* and *Agathobacter*. Compared with the SLE-nd group, the SLE-d group had elevated serum levels of IL-2 and IL-6 and decreased BDNF. Interestingly, abundance of the genus *Faecalibacterium* and *Roseburia* was negatively correlated with IL-6, abundance of the genus *Roseburia* was negatively correlated with IL-2, and abundance of the genus *Bacteroides* was positively correlated with IL-2.

**Conclusion:**

This study identified specific fecal microbes and their metabolites that may participate in the development of SLE-d. Our findings provide a new perspective for improving depression in SLE patients by regulating the gut–brain axis.

## Introduction

Systemic lupus erythematosus (SLE) is an autoimmune condition with no recognized etiology. The mechanisms of SLE may be traced back to an intricate combination of genetic, endocrine, infectious, immunological, and environmental variables. Accumulating evidence demonstrates that SLE patients are prone to depression and anxiety and other psychological manifestations (Kozora et al., 2006; Waldheim et al., 2013). SLE patients are twice as likely to experience depression as average healthy people (Bachen et al., 2009). Depression in SLE patients, in addition to social and psychological factors and the characteristics of the disease itself (such as a long course of the disease and illness repeatedly, steroid hormone dose, and physical symptoms), is also closely related to the imbalance of gut microbiome; thus, gut microbiome may be the cause of depression in SLE.

As reported previously (Mu et al., 2015), SLE is regulated by microorganisms and diet. In addition, it has been shown that the gut microbiome of SLE patients and mouse models have quantitative and structural abnormalities compared with the microbiota of other healthy controls (Rahbar Saadat et al., 2019). The MRL/Mp-Faslpr (MRL/lpr) mouse model of classical SLE has been used to explore the dynamics of gut microbiome in autoimmune lupus (Mu et al., 2015). Compared with age-matched healthy mice, decreased abundance of *Lactobacilli* and increased abundance of *Clostridial* species (Lachnospiraceae), together with increased bacterial diversity, were found in young female lupus-affected mice, suggesting that gut microbiome imbalance is linked to SLE development.

The gut microbiome is a complex ecology. The human body has 10^13^ to 10^14^ bacteria, the vast majority of which belong to the two eminent phyla Firmicutes and Bacteroides (Sears and Garrett, 2014). Recent findings suggest that alterations in the composition of an individual’s gut microbiome may contribute to the development of depression by altering the connection between the gut–brain axis and mucosal immunity (Naseribafrouei et al., 2014). In addition, the influence of gut microbiome on the bidirectional communication between the mucosal immune system and the gut–brain axis is manifested in the correlation between intestinal autonomic nerves, vagus nerves, and the central nervous system, as well as the relationship between the imbalance of gut microbiome and autoimmunity. Moreover, as a neurotrophic factor, brain-derived neurotrophic factor (BDNF) is important for the development and differentiation of brain cells, neurogenesis, and synaptic rewiring, as well as for the neurophysiological causes of depression (Brunoni et al., 2008; aan het Rot et al., 2009). In addition, activated inflammatory systems, including cytokines (IL-2 and IL-6), play a crucial role in the pathogenesis of depression (Maes et al., 1990; Liu et al., 2012). They may be involved in a two-way communication between the mucosal immune system and the gut–brain axis. However, no study has yet confirmed the correlation between depression, gut microbiome, and the gut–brain axis in SLE patients.

Metabolomics is a promising method for identifying metabolite biomarkers in clinical diagnostics (Kishikawa et al., 2021). In metabolomics, it is generally agreed that non-targeted metabolomics plays an essential role. Compared with focused metabolomics, this method requires the collection of metabolomics data, which is both more detailed and complete. In this study, the gut microbiome of SLE patients was compared with that of healthy controls, and structural and metabolic differences between the two groups were investigated. This study highlights the imbalance in gut microbiome and associated metabolites in SLE patients with depression.

## Materials and methods

### Participants

The Department of Immunology and Rheumatology, Qingdao Municipal Hospital, selected 38 SLE patients as stipulated by the 1997 American College of Rheumatology (ACR) categorization criteria from September 2020 to May 2022. Thirty-two healthy Chinese individuals were recruited as healthy controls (HC). Only females were recruited for this analysis because of the high prevalence of SLE in women (female:male=9:1). This study was approved by the ethics committee of Qingdao Municipal Hospital, Qingdao University. Each subject provided informed consent to participate in the study. All subjects were from different regions of the Shandong Province of China. The Self-Rating Depression Scale (SDS) was used to measure depression in all subjects. The characteristics of all the subjects are shown in **Table 1**. None of the subjects had ever had a probiotic diet or received antibiotic treatment within the previous 8 weeks. None of the subjects had any other autoimmune diseases, hypertension, diabetes, severe mental illness, inflammatory bowel disease, other inflammatory diseases, autoimmune and infectious disorders, or malignant tumors. Based on the SLEDAI-2000 score, all patients were inactive or mildly active, and the current treatment regimen was maintenance of hormones at a low dose (<10 mg) or hydroxychloroquine alone. Subjects with a past history of psychiatric depression before being diagnosed with SLE and incomplete or missing medical records were excluded.

**Table 1:** Characteristics of the subjects.

	SLE-d (n = 21)	SLE-nd(n=17)	HC (n = 32)	*P*-value
Female	100%	100%	100%	——
Age, years	39.66 ± 13.20	43.58 ± 14.47	38.51 ± 14.28	*P* = 0.35
BMI, kg/m^2^	21.08 ± 1.72	21.77 ± 1.96	22.17 ± 1.98	*P* = 0.17
ESR, mm/h	43.00 ± 28.00	24.41 ± 12.12	12.51 ± 3.54	*P* = 0.000
CRP, mg/L	11.83 ± 2.40	7.86 ± 2.82	3.20 ± 4.12	*P* = 0.001
24-hour urinary protein quantity, g	0.56 ± 0.12	0.49 ± 0.07	0.47 ± 0.13	*P* = 0.36
Creatinine, umol/L	65.96 ± 12.73	63.27 ± 11.21	62.08 ± 10.97	*P* = 0.22
GFR, ml/min•1.73cm^2^	108.83 ± 5.44	112.32 ± 5.25	114.87 ± 6.13	*P* = 0.19
TG, mmol/L	1.21 ± 0.63	1.07 ± 0.51	1.18 ± 0.60	*P* = 0.21
Cholesterol	4.41 ± 0.54	4.26 ± 0.64	4.36 ± 0.72	*P* = 0.57
AST, U/L	22 ± 1.31	19 ± 1.21	23 ± 1.09	*P =*0.44
ALT, U/L	19 ± 4.11	16 ± 4.08	17 ± 5.60	*P*= 0.056
SDS	≥50	<50	<50	——

BMI, body mass index; ESR, erythrocyte sedimentation rate; CRP, C-reactive protein; GFR, glomerular filtration rate; TG, triacylglycerol; AST, aspartate transaminase; ALT, alanine aminotransaminase; SDS, Self-Rating Depression Scale.

### Data and sample collection

This study evaluated the depressive state in SLE patients using SDS created by Duke University Professor William W.K. Zung, which can accurately capture the subjective experiences of patients with depression. The scale contains 20 items (10 positive and 10 negative) that reflect the subjective feelings of depression. Each item is split into four grades according to the frequency of symptoms. SDS is mainly applicable to adults with depressive symptoms and has high sensitivity (Dunstan et al., 2017). It is commonly used during psychological counseling for outpatients, psychiatric outpatients, and inpatients. Using a standard score of 50 as the cutoff point, values above this threshold were considered indicative of the presence of depression; SLE patients with SDS scores of ≥50 were included in the SLE patients with depression (SLE-d) group (n=21), whereas those with SDS scores of <50 were included in the SLE patients without depression (SLE-nd) group (n=17). Patients in the HC group (n=32) were excluded from the depression group with an SDS score of <50.

Stool and serum samples were collected from patients on the same day as the scale data were collected. Fecal samples were collected in sterile tubes. After collecting and centrifuging at 2400 rpm for 10 min at four temperature venous blood samples from fasting patients, the supernatant was removed and placed into an Eppendorf tube. Serum samples with hemolysis were excluded from this study. All stool and serum samples were stored in a refrigerator at −80°C.

### Fecal DNA extraction and 16S rRNA gene sequencing

The TGuide S96 Magnetic Soil/Stool DNA Kit (Tiangen Biotech (Beijing) Co., Ltd., Beijing, China) was used to recover genomic DNA from the fecal samples. Polymerase chain reaction (PCR) was used to amplify the highly variable region of the 16S rRNA gene sequence known as v3-v4 (388F-primer: 5′ACTCCTACGGGAGGCAGCA-3′ and 806R-primer: 5′-GGACTACHVGGGTWTCTAAT-3′), 50 ng+−20% of genomic DNA, 0.3 μL of VnF (10 μM), 0.3 μL of VnR (10 μM), 5 μL of KOD FX Neo Buffer, 2 μL of dNTP (2 mM), 0.2 μL KOD FX Neo, and ddH2O to 10 μL for the first cycle of PCR, the PCR conditions were initial denaturation at 95°C for 5 min, followed by 25 cycles of denaturation at 95°C for 30 s, 50°C for 30 s, 72°C for 40 s, 72°C for 7 min, and 4°C ∞. The following steps involved a second cycle of PCR, 5 μL of target region PCR purified product, 2.5 μL of 2 μM MPPI-a, 2.5 μL of 2 μM MPPI-b, and 10 μL of 2×Q5 HF MM. The PCR conditions were 98°C for 30 s, followed by 10 cycles of denaturation at 98°C for 10 s, 65°C for 30 s, 72°C for 30 s, and 72°C for 5 min; equal amounts of samples were mixed according to the concentration of PCR products, the mixed PCR products were then purified using the OMEGA DNA Gel Extraction Kit and recovered using the Monarch DNA Gel Recovery Kit, and the Illumina Novaseq 6000 sequencing technology was used to sequence the generated material. Raw reads were filtered using Trimmomatic after Illumina sequencing (version 0.33). Cutadapt (version 1.9.1) was used to determine the primer sequences and obtain tags devoid of primer sequences. Usearch was used to perform double-endian sequence splicing (version 10). Chimeric sequences were identified and eliminated, and the final effective tags were produced using UCHIME (version 4.2). The final effective tag sequences with 97% sequence identity were jointly classified as an operational taxonomical unit (OTU). By searching against the SILVA database (Release 138.1, http://www.arb-silva.de), the feature sequence was annotated using a simple Bayesian classifier to obtain taxonomic information. In this study, alpha diversity was determined using the QIIME2 software application to compare the richness and diversity of each sample, and QIIME was used to explore beta diversity to examine the comparability of species diversity among samples. The R programming language was used to analyze the principal coordinates. Linear discriminant analysis (LDA) effect size (LEfSe) was used to identify the characteristics of each category.

### Ultra-high-performance liquid chromatography-quadrupole time-of-flight mass spectrometry

The Waters UPLC Acquity I-Class PLUS system, in conjunction with the Waters UPLC Xevo G2-XS QTof and Acquity UPLC HSS T3 1.8 m 2.1×100 mm chromatographic column, was used to conduct the metabolism analysis. Solvents A (water with 0.1% formic acid) and B (acetonitrile with 0.1% formic acid) were used as mobile phases. Elution gradient program for a 15-min run was set as follows: flow rate: 0.4 mL/min; 98% A 2% B from 0 to 0.25 min; 2% A 98% B from 0.25 to 10 min; 98% A 2% B from 10 to 13 min; and 98% A 2% B from 13.1 to 15 min. For its capacity to gather primary and secondary mass spectrometry data in the MSe mode under the supervision of the acquisition software program, the Waters Xevo G2-XS QTof High-resolution MS was employed (MassLynx version 4.2, Waters). The ESI source had the following characteristics: ion source temperature: 150°C; desolvent gas temperature: 500°C; blowback flow rate: 50 L/h; desolvent gas flow rate: 800 L/h; capillary voltage: 2000 V (positive ion mode) or −1500 V (negative ion mode); and taper hole voltage: 30 V. Peak extraction and peak alignment were part of the initial processing performed on the raw data using Progenesis QI software. For identification and theoretical fragment identification, the BMK self-built database and online METLIN database were employed. The mass number discrepancy was <100 ppm. Student’s t-test was used to analyze the data and draw conclusions regarding differences in metabolite concentrations between the groups. The metabolites were screened based on multiple differences, P-value from t-test, and variable importance in the projection (VIP) of the orthogonal projections to latent structures discriminant analysis (OPLS-DA), an orthogonal partial least squares discrimination analysis, model (FC>1, *P*< 0.05, and VIP>1).

### Enzyme-linked immunosorbent assay

As stated before, serum samples were collected from participants in the SLE-d, SLE-nd, and HC groups. According to the manufacturer’s recommendations, serum BDNF, IL-2, and IL-6 levels were assessed using a standardized ELISA kit (Abcam).

### Statistical analysis

Statistical analysis was performed using SPSS version 23.0. The Quantitative data with a normal distribution were expressed as the mean ± standard deviation. The data were compared among the three groups using analysis of variance, the counting data were expressed as the counting ratio for continuous variables, and data comparisons were conducted using Pearson’s chi-square test. Open data were subjected to the rank-sum test. Alpha and beta diversity studies of the genetic sequencing data were performed using R programming language. To identify important differences across the groups, Wilcoxon rank-sum test and Metastats analysis was employed. Connections between variables were evaluated using hierarchical clustering, principal coordinate analysis (PCoA), LEfSe analysis, and Spearman’s rank correlation studies.

## Results

### Sequencing data and the alpha diversity index

**Figure 1:**
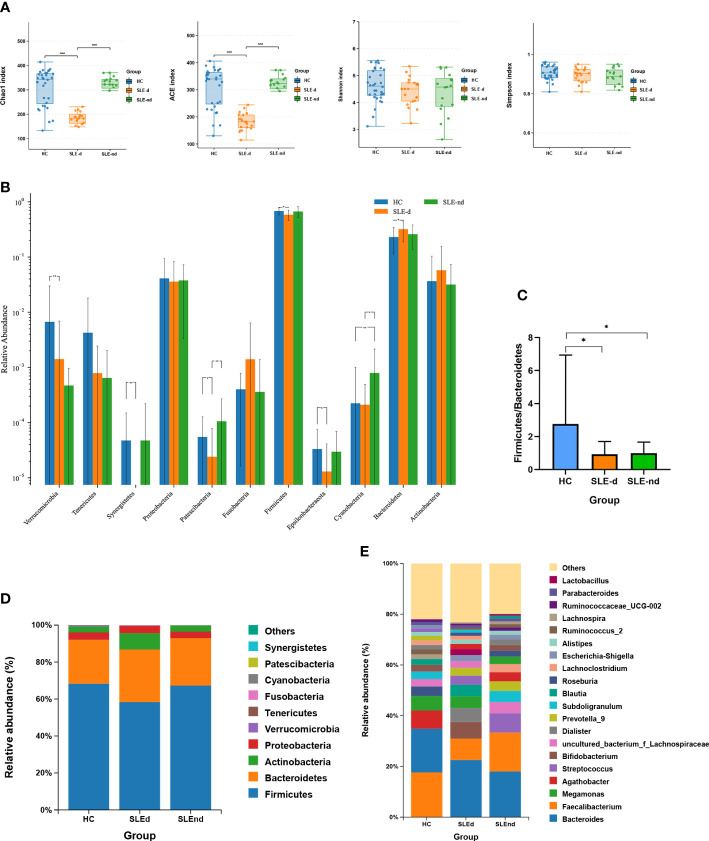
Microbial structures of the three groups. **(A)** Ace, Chao, Shannon, and Simpson indexes of the different groups, ***P < 0.001. **(B)** Significant differences in bacteria in phylum levels among the three groups, *P < 0.05, **P < 0.01. **(C)** Comparison of the three groups of Firmicutes/Bacteroides phylum ratios, *P < 0.05. **(D)** Relative abundance of gut microbes at phylum levels in the three groups. **(E)** Relative abundance of gut microbes at the genus level.

Overall, 372 OTUs in the SLE-d group, 456 OTUs in the SLE-nd group, and 464 OTUs in the HC group were collected by detecting fecal samples from the three groups (**Figure S1**). While the ACE (*t*=0.15, *P*=0.8811), Chao1 (*t*=0.04, *P* = 0.9667), Shannon (*t*=1.12, *P*=0.2045), and Simpson (*t*=1.572, *P*=0.1228) indices did not reveal any differences between the SLE-nd and HC groups, the ACE (*t*=7.23, *P*=0.000) and Chao1 (*t*=7.16, *P*=0.000) indices showed that the fecal microbial richness in SLE-d patients was significantly lower than that in the HC group (**Figure 1A**).

### Altered microbiota composition among groups

The Wilcoxon rank-sum test was applied to compare the fecal microbial compositions among three groups at the phylum level. The SLE-nd group had significantly higher relative abundances of Cyanobacteria (χ² =9.64, P=0.008) than the HC group, and the SLE-d group had a lower abundance of Patescibacteria (P=0.009) and Cyanobacteria (P=0.035) than the SLE-nd group (**Figure 1B**). Compared with the HC group, SLEd patients showed a lower relative abundance of Firmicutes (P=0.013), higher abundance of Bacteroidetes (P=0.03), decreased ratio of Firmicutes to Bacteroidetes (F/B) (P<0.05; **Figure 1C**), and significantly decreased relative abundance of Verrucomicrobia (χ² =16.91, P=0.000), Synergistetes (χ² =11.07, P=0.004), Epsilonbacteraeota (P=0.026), and Patescibacteria (P=0.046); At the phylum levels, the three dominant phyla in the SLE-d group were Firmicutes (0.583 ± 0.0266), Bacteroidetes (0.319 ± 0.0287), and Actinobacteria (0.0577 ± 0.0213); in the SLE-nd group, these were Firmicutes (0.669 ± 0.0361), Bacteroidetes (0.259 ± 0.0299), and Proteobacteria (0.0378 ± 0.00835); and in the HC group, these were Firmicutes (0.681 ± 0.0184), Bacteroidetes (0.23 ± 0.0205), and Proteobacteria (0.041 ± 0.0094) (**Figure 1D**).

Metastats analysis was used to compare the fecal microbial compositions between the two groups at the class, family, order, and genus levels. After the *P*-value correction, there were no differences in the abundance of bacteria between the SLE-nd and HC groups. Compared with the SLE-nd group, the relative abundance of Synergistetes (*P*=0.017) was decreased in the SLE-d group at the class level (**Figure S2**), whereas at the family level, SLE-d patients showed a lower relative abundance of Brevibacteriaceae (*P*=0.007), Moraxellaceae (*P*=0.007), Planococcaceae (*P*=0.007), Porphyromonadaceae (*P*=0.007), Synergistaceae (*P*=0.007), Muribaculaceae (*P*=0.007), and Caulobacteraceae (*P*=0.04) and higher abundance of Staphylococcaceae (*P*=0.007), Pseudomonadaceae (*P*=0.007), Propionibacteriaceae (*P*=0.018; **Figure S3**). At the order level, there was a decreased relative abundance of Pseudomonadales (*P*=0.014) and Synergistale (*P*=0.014) and an increased relative abundance of Propionibacteriales (*P*=0.028; **Figure S4**).

At the genus level, in the order of abundance, the most predominant gut microbiota were *Bacteroides* (0.238 ± 0.0314), *Faecalibacterium* (0.094 ± 0.02), and *Bifidobacterium* (0.0419 ± 0.0181) in the SLE-d group; *Bacteroides* (0.187 ± 0.0278), *Faecalibacterium* (0.15 ± 0.0247), and *Streptococcus* (0.0705 ± 0.0420) in the SLE-nd group; and *Faecalibacterium* (0.169 ± 0.0155), *Bacteroides* (0.163 ± 0.0179), and *Agathobacter* (0.0692 ± 0.0116) in the HC group (**Figure 1E**). Among the predominant genera, *Faecalibacterium* (*P*=0.031) and *Agathobacter* (*P*=0.021) decreased in the SLE group. Compared with the HC group, the SLE-d group had decreased relative abundances of *Faecalibacterium* (*P*=0.01), *Agathobacter* (*P*=0.007), *Roseburia* (*P*=0.016), *Subdoligranulum* (*P*=0.001), and *Lachnospira* (*P*=0.003) and increased relative abundance of *Bacteroides* (*P*=0.047). The abundances of *Faecalibacterium* and *Roseburia* were lower in the SLE-nd group than in the HC group (P<0.05) and were significantly lower in the SLE-d group than in the SLE-nd group (P<0.05).

### Beta diversity analyses among groups

**Figure 2:**
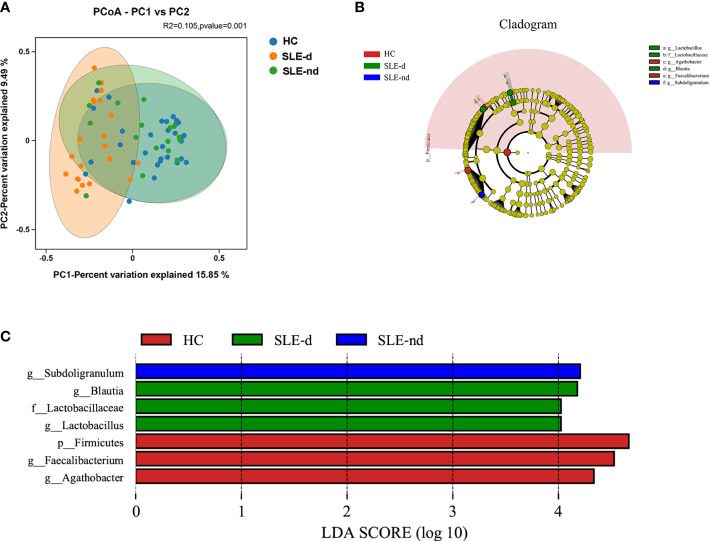
**(A)** At the OTU level, the principal coordinates analysis (PCoA) revealed fecal microbiota compositions in the three groups based on the Bray-Curtis distance. *R^2^* = 0.105, *P* = 0.001. **(B)** Cladogram from phylum to genus levels of the LEfSe analysis. **(C)** Histogram of the LDA scores (LDA >4) for different representative genera in HC, the SLE-d group, and the SLE-nd group.

At the OTU level, the beta diversity difference using the Bray-Curtis distance was shown in the PCoA analysis. A comparison of the three groups showed statistically significant differences (*P*=0.001; **Figure 2A**).

### LEfSe analysis among groups

Cladograms from all three groups at the phylum to genus level (**Figure 2B**) and unique fecal microbial taxa were isolated using the LEfSe method. *g-Subdoligranulum* (LDA: 4.22; *P*=0.002) was the representative flora in the SLE-nd group; *g-Blautia* (LDA: 4.15; *P*=0.002), *g-Lactobacillus* (LDA: 4.12; *P*=0.011), and *f-Lactobacillaceae* (LDA: 4.12; *P*=0.011) in the SLE-d groups; and g-*Faecalibacterium* (LDA: 4.58; *P*=0.023), *p-Firmicutes* (LDA: 4.66; *P*=0.01), and *g-Agathobacter* (LDA: 4.33; *P*=0.009) in the HC group (**Figure 2C**). LEfSe analysis revealed potential biomarker flora with statistically significant differences.

### Overall metabolomics analyses of fecal samples

**Figure 3:**
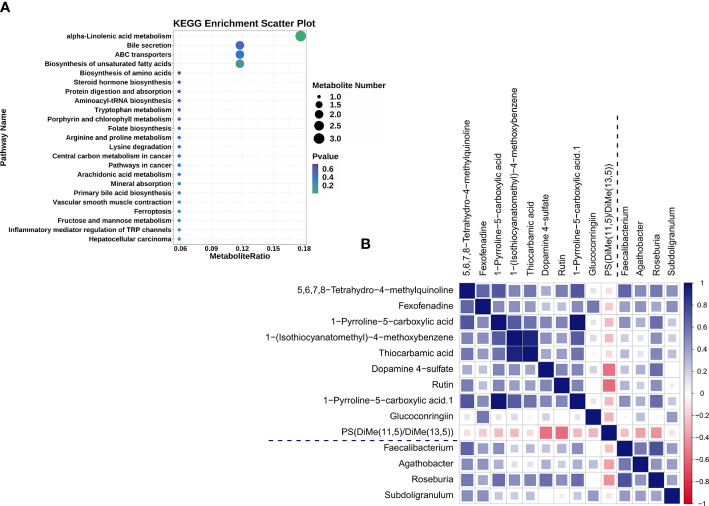
**(A)** The 19 differential metabolites for SLE-d group compared with HC patients were enriched in KEGG pathways. **(B)** Heat map summarizing the correlation of altered gut microbiota genera and fecal metabolites between the SLE-d and HC groups.

UHPLC-QTOF-MS was used to gather fecal metabolomic information from the SLE-d and HC groups. With a Q2 of 0.557 and an R2 of 0.992, OPLS-DA (**Figure S5**) showed a substantial metabolic difference between the two groups. These results demonstrate that the proposed model is both robust and reliable. Of the 236 metabolites that were identified as altered in the SLE-d group, 30 metabolites increased and 206 decreased (VIP>1.00, P*<*0.05). The metabolites with the highest VIPs were ganglioside GA2 (d18:1/18:0), austroinulin, 15-oxo-lipoxin A4, PS (14:0/18:0), linamarin, arginyl-tyrosine, (S, E)-zearalenone, and PC (20:4(5Z,8Z,11Z,14Z)/P-18:1(9Z)). There were 19 metabolites with the KEGG ID annotation: the levels of 3-α, 11-β, 21-trihydroxy-5β-pregnan-20-one, stearidonic acid, L-proline, 2-aminomuconic acid, 6-carboxy-5,6,7,8-tetrahydropterin, menthol, D-urobilinogen, alpha-linolenic acid, 20-hydroxyeicosatetraenoic acid, jasmonic acid, 4-hydroxynonenal, rutin, mannitol 1-phosphate, colistin, and fexofenadine were upregulated in the fecal samples of SLE-d patients; in contrast, the levels of linamarin, cholic acid, tylosin, and adrenic acid were downregulated in these patients (**Table 2**). The differential metabolites of HC vs. SLE-d were mainly involved in alpha-linolenic acid metabolism (stearidonic acid, alpha-linolenic acid, jasmonic acid), biosynthesis of unsaturated fatty acids (adrenic acid, alpha-linolenic acid), ATP-binding cassette transporters (l-proline and colistin), and bile secretion (fexofenadine, cholic acid). In addition, they included tryptophan metabolism, aminoacyl-tRNA biosynthesis, and lysine degradation pathways (**Figure 3A**).

**Table 2:** Fecal identified part differential metabolites between SLE-d patients and healthy controls.

Name	(HC/SLE-d) log2FC	VIP	*P*-value	Regulated
Ganglioside GA2 (d18:1/18:0)	-2.400393743	4.04E-06	3.033163387	down	
Austroinulin	-0.874904737	3.38E-06	2.991213806	down	
15-Oxo-lipoxin A4	-0.700846105	3.49E-05	2.85316749	down	
PS (14:0/18:0)	-0.724252511	9.71E-05	2.707937169	down	
Linamarin	2.675458943	0.000864324	2.639760401	up	
Arginyl-Tyrosine	2.706491525	0.001229384	2.581733509	up	
(S, E)-Zearalenone	2.742166905	0.00139812	2.559166235	up	
6-Propyl-2-thiouracil	3.522012406	0.002235105	2.466905832	up	
PC (20:4(5Z,8Z,11Z,14Z)/P-18:1(9Z))	-1.363051229	0.000561326	2.46156213	down	
(E, E)-11,13-Octadecadien-9-ynoic acid	-1.092829553	0.001244428	2.436617818	down	
Erysothiopine	-5.666067031	0.001837387	2.419296248	down	
Menthone 1,2-glyceryl ketal	-0.578225155	0.001221059	2.413031641	down	
1-O-Hexadecyl-2-O-dihomogammalinolenoylglycero-3-phosphocholine	-0.927749032	0.00068067	2.412202986	down	
Domoic acid	1.74983097	0.003220183	2.384571032	up	
2-Hydroxy-6-tridecylbenzoic acid	-1.922159134	0.001908139	2.383326964	down	
Ligustilide	-1.034272957	0.001649343	2.381769736	down	
Homoveratric acid	-10.18920453	0.001711178	2.378060036	down	
Miglustat	-0.909259696	0.000917894	2.370109383	down	
alpha-Methylphenylalanine	-1.176078254	0.004345598	2.363382932	down	
Dihydrojasmonic acid	-1.111756661	0.001304417	2.332352457	down	
7’,8’-Dihydro-8’-hydroxycitraniaxanthin	-12.77929108	0.005011067	2.318120808	down	
PC (14:0/18:3(6Z,9Z,12Z))	-1.373223738	0.001820353	2.295527343	down	
(S)-[8]-Gingerol	-1.321618735	0.002951399	2.265997477	down	
L-dopachromate	1.563221933	0.00350275	2.265348785	up	
xi-4-Hydroxy-4-methyl-2-cyclohexen-1-one	-0.82490826	0.001769706	2.260333367	down	
Mepenzolate	-0.795208212	0.004110312	2.254833349	down	
Cotinine N-oxide	-1.184455044	0.002684743	2.246068365	down	
12-hydroxyicosanoic acid	-1.716331785	0.003285634	2.245450558	down	
Riesling acetal	-1.011354721	0.002263321	2.223040281	down	
N-Arachidonoyl tyrosine	1.084110479	0.00794091	2.220237064	up	
Ganglioside GA2 (d18:1/18:0)	-2.400393743	4.04E-06	3.033163387	down	

### Correlation analysis of gut microbiome and fecal metabolites

To explore the correlation between the top 30 differential microbiomes and the top 30 differential fecal metabolomes, we performed Spearman correlation analysis (**Figure S6**). The altered bacterial genera were correlated with a series of differential metabolites (**Figure 3B**). For example, 5,6,7,8-tetrahydro-4-methylquinoline, fexofenadine, and 1-pyrroline-5-carboxylic acid were positively correlated with *Faecalibacterium*; 5,6,7,8-tetrahydro-4-methylquinoline and fexofenadine were positively correlated with *Agathobacter*; 1-(isothiocyanatomethyl)-4-methoxybenzene, thiocarbamic acid, dopamine 4-sulfate, 5,6,7,8-tetrahydro-4-methylquinoline, rutin, and 1-pyrroline-5-carboxylic acid were positively correlated with *Roseburia*; fexofenadine, 5,6,7,8-tetrahydro-4-methylquinoline, and glucoconringiin were positively correlated with *Subdoligranulum*; and PS(DiMe(11,5)/DiMe(13,5)) was negatively correlated with *Roseburia*.

### Measurement of serum BDNF, IL-2, and IL-6 levels by ELISA

**Figure 4:**
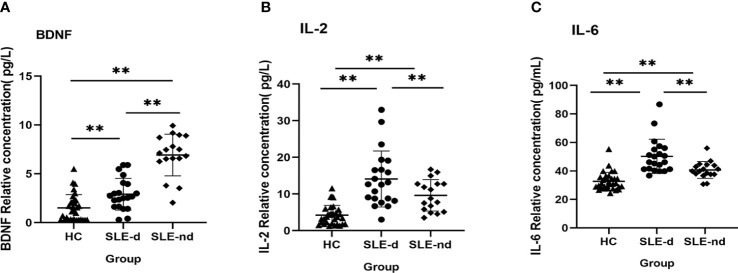
**(A)** Serum BDNF levels in different groups. **P < 0.01. **(B)** Serum IL-2 levels in different groups, **P < 0.01. **(C)** Serum IL-6 levels in different groups, **P < 0.01.

To compare the inflammatory state and serum BDNF levels of SLE-d patients with those of SLE-nd patients and HC, we measured serum the levels of IL-2, IL-6, and BDNF in the samples from all patients. The findings showed that the SLE-d group had significantly lower serum BDNF levels than the SLE-nd group (2.9 ± 0.354 vs. 6.92 ± 0.517, *P* = 0.000), and the HC group had lower serum BDNF levels than the SLE-d group (2.9 ± 0.354 vs. 1.502 ± 0.241, *P* = 0.001) (**Figure 4A**). The levels of serum IL-2 (4.22 ± 0.468 vs. 9.62 ± 1.04 vs. 14.08 ± 1.665, *P <*0.001) (**Figure 4B**) and IL-6 (32.69 ± 1.102 vs. 40.67 ± 1.433 vs. 50.24 ± 2.632, *P <*0.001) (**Figure 4C**), as compared with the HC group, were significantly higher in the SLE-nd group but lower in the SLE-nd group than in the SLE-d group.

### Correlations between gut microbes and serum BDNF, IL-2, and IL-6 levels

**Figure 5:**
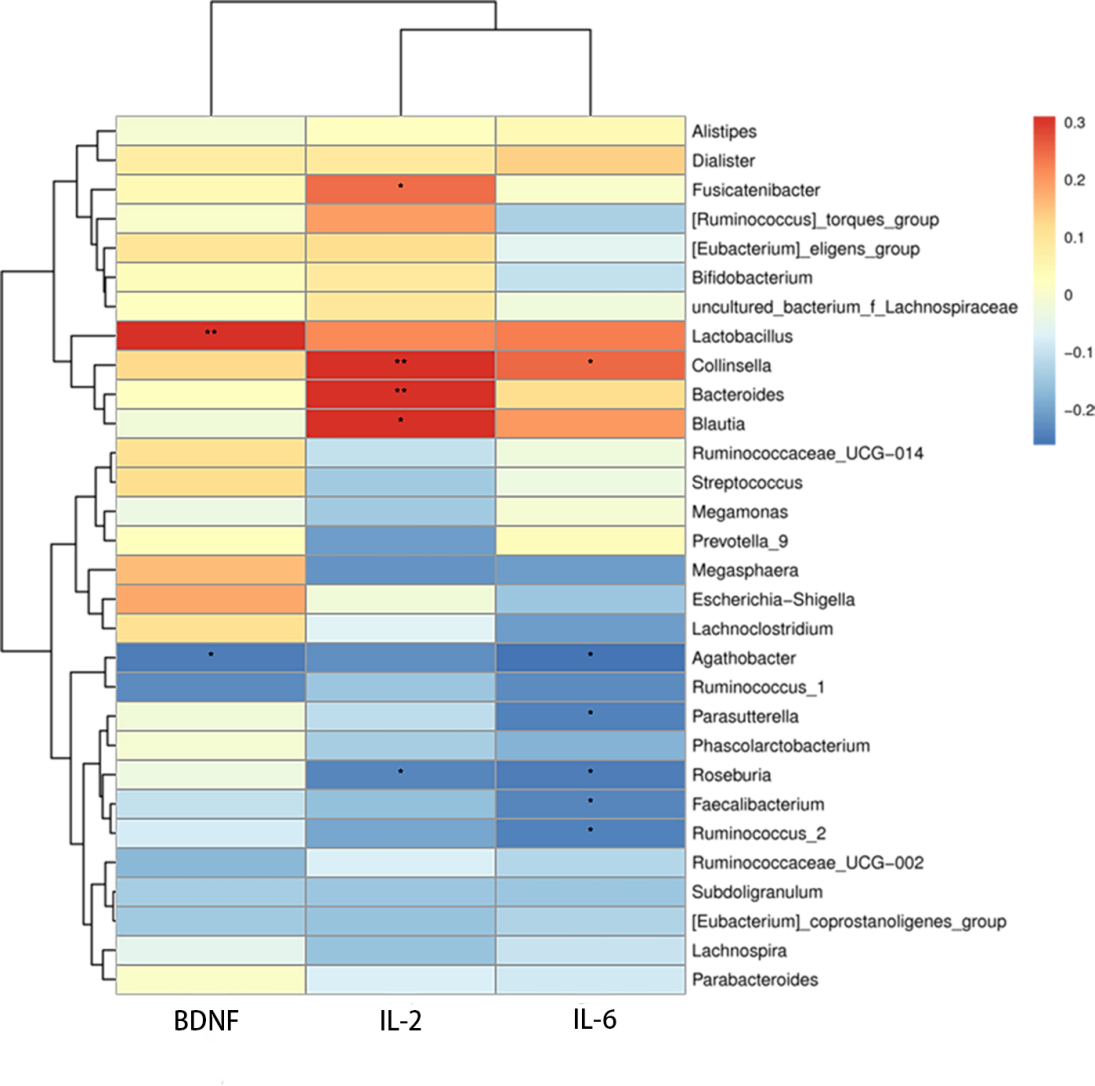
Heatmap of correlations between microbial abundances of genera and serum IL-2, IL-6, and BDNF levels, *P <0.05, **P < 0.01.

Additionally, we studied the relationships among BDNF, serum autoinflammatory factors, and the relative abundance of various bacterial genera. The relative abundance of *Agathobacter* (*r* =0.284, *P* =0.017) was significantly inversely correlated with serum BDNF level, and the relative abundance of *Lactobacillus* (*r* =0.242, *P* =0.043) was positively correlated with serum BDNF level. Significant positive relationships were discovered between serum IL-2 and the bacteria *Bacteroides* (*r* =0.306, *P* =0.009), *Blautia* (*r* =0.305, *P* =0.01), *Fusicatenibacter* (*r* =0.242, *P* =0.043), and *Collinsella* (*r* =0.31, *P* =0.009). While there was a negative correlation between *Roseburia* (*r* =-0.235, *P* =0.049) and serum IL-2, *Agathobacter* (*r* =-0.261, *P* =0.029), *Faecalibacterium* (*r* =-0.237, *P* =0.047), *Roseburia* (*r* =-0.247, *P* =0.038), *Ruminococcus-2* (*r* =-0.235, *P* =0.049), *Parasutterella* (*r* =-0.235, *P* =0.049), and *Ruminococcus-2* (*r* =-0.24, *P* =0.045) all showed negative correlation with serum IL-6, whereas *Collinsella* (*r* =0.249, *P* =0.037) showed a positive correlation with serum (**Figure 5**).

## Discussion

A previous study (He et al., 2020) revealed that abnormalities in the gut microbiome and related metabolites may affect SLE development, and depression is widespread among SLE patients (Monahan et al., 2021). However, the underlying causes and pathophysiology of this elevated risk are not well understood. This study analyzed the relationship between psychiatric symptoms in SLE patients and their gut microbiome, which may encourage more research into the underlying mechanism.

Here, we compared the fecal microbiota across a variety of population subsets and found that SLE patients with depression have a distinct gut microbiome composition compared with their counterparts without depression. In SLE patients, the relative abundance of Bacteroidetes increased, whereas that of Firmicutes decreased. These findings are consistent with those reported in a previous study (Hevia et al., 2014). This may be related to oxidative phosphorylation and the enhanced metabolism of metabolic pathways in the intestines of these patients (Hevia et al., 2014). Previous studies on patients with various disorders such as type 2 diabetes, obesity, Crohn’s disease, and irritable bowel syndrome reported an imbalance between Firmicutes and Bacteroidetes in the human gut (Turnbaugh et al., 2006; Larsen et al., 2010; Man et al., 2011; Liu et al., 2020); however, another study found no difference in the F/B ratio between SLE patients with active disease and HC (Luo et al., 2018). In addition, we discovered that the abundance of bacteria of the phylum Bacteroidetes and genus *Bacteroides* was significantly increased, whereas that of bacteria of the phyla Firmicutes, Verrucomicrobia, Synergistetes, Epsilonbacteriaeota, and Patescibacteria and the genera *Faecalibacterium*, *Agathobacter*, *Roseburia*, *Subdoligranulum*, and *Lachnospira* was significantly decreased in SLE-d patients compared with HC. A study on multiple depression rat models found that the Muribaculaceae family was associated with abnormal lipid and amino acid metabolism (Liu et al., 2022). The recovery of depression in chronic variable stress–induced rats is attributed to an increase in the abundance of Pseudomonadaceae in the gut tract of mice treated with antibiotics (Yu et al., 2022). Contrary to our conclusion, a study found higher abundance of bacteria of the family Moraxellaceae in the oral cavity of SLE patients than in HC (Li et al., 2020). An increased relative abundance of Planococcaceae and decreased relative abundance of Moraxellaceae, Staphylococcaceae, and Propionibacteriaceae were reported in the upper respiratory tract of patients with granulomatosis with polyangiitis and rheumatoid arthritis (Lamprecht et al., 2019). The discrepancies in the results could be explained by individual variances in the study population. Other differences in microbes have not been previously studied in SLE patients with depression; therefore, further studies on the function of certain microorganisms are warranted to explore the role of the flora in SLE patients with depression. The relationship between dysbiosis of gut microbiome and intestinal inflammation has been established (Hughes et al., 2017). Intestinal immunological problems and inflammation have been observed in individuals with depression (Schiepers et al., 2005; Rook and Lowry, 2008; Lindqvist et al., 2017), and comparable microbiota abnormalities have been observed in animal models of depression (O’Mahony et al., 2009; Park et al., 2013; Yu et al., 2017). Compared with HC, individuals with major depressive disorder have higher levels of Actinobacteria, Bacteroidetes, and Proteobacteria, but a previous study found that these patients have higher levels of *Enterobacteriaceae* and *Allistipes* and lower levels of *Faecalibacterium* (Jiang et al., 2015). Some studies have shown that the favorable health effects observed in SLE patients may be due to short-chain fatty acids (SCFAs) produced by *Bacteroidetes* during fiber fermentation (Leung et al., 2016; Canfora et al., 2019). Butyrate has been the subject of considerable research (Ríos-Covián et al., 2016; Stilling et al., 2016; Li et al., 2021). SCFAs produced by the microbiota of the large intestine are vital for regulating systemic immune responses and influencing the intestinal environment. Previous research has shown that butyrate alters T cell function by activating histone acetyltransferase or inhibiting histone deacetylase, thereby lowering the inflammatory response (Koh et al., 2016). Indirectly or directly, it may affect brain function and gut–brain connections *via* immunological, endocrine, vagal, and other humoral mechanisms (Stilling et al., 2016). *Faecalibacterium*, *Roseburia*, and *Lachnospira*, all of which are capable of generating butyrate, were shown to be deficient in SLE-d samples. This decreases SCFAs production, which may trigger an inflammatory reaction. Gut microbiome composition may play a critical role in the interaction between lupus and the brain, as evidenced by our data, which indicated significant variations among the three groups.

The metabolome of clinical participants’ fecal and serum samples from SLE patients showed altered metabolites and metabolic pathways, according to the available data on the disease. In a recent study, 55 substantially altered metabolites were regularly detected in the stool samples of 21 SLE patients and 10 healthy controls. Serum metabolome data showed that several metabolites, particularly SCFAs such as endocrinol and bile acids, underwent considerable changes (He et al., 2020). Pentanoate has been linked to autoimmune disorders and inflammation as it boosts IL-10 production and suppresses Th17 cells (Luu et al., 2019). Ceramides, trimethylamine N-oxide, xanthine, and hydrocortisone are novel biomarkers (Li et al., 2019). The tryptophan catabolites xanthurenic acid and kynurenic acid were among the 23 metabolites found in SLE patients in a different study that used UHPLC-MS for fecal metabolomics; other abnormalities included aminoacyl-tRNA biosynthesis, thiamine metabolism, nitrogen metabolism, and cyanoamino acid metabolism (Zhang et al., 2019). Additionally, studies on sad humans and depression in animals have recently discovered anomalies in tryptophan and bile acid metabolism (Su et al., 2014). Our results partially overlap with those described above. Tryptophan is a biosynthetic precursor of many microbes and metabolites. Intestinal microbiota may participate in neuropsychiatric diseases by regulating the validity of circulating tryptophan through direct and indirect effects (Deng et al., 2021). As a tryptophan metabolite, 5-hydroxytryptamine is synthesized by enterochromaffin cells. The generated signal can be sent to external neurons and specific receptors through the intestine to participate in the microbiota gut–brain axis (Deng et al., 2021). In the correlation analysis, we focused on metabolites associated with key differential genera and found a correlation between downregulated fexofenadine metabolites and decreased levels of *Faecalibacterium*, *Agathobacter*, and *Subdoligranulum* genera in the SLE-d group, which seems to be involved in the bile secretion pathway and may be used as a potential marker. Here, we analyzed the fecal metabolic spectrum between the groups and found that the fecal metabolic spectrum of SLE patients with depression was different from that of HC. Some metabolic pathways overlap with those of SLE and depression. Expounds the relationship between the microbiome and fecal metabolism and requires larger cohort studies to explore the causal relationship between the two. These results suggest increased fecal metabolism preceding the onset of depression, which is likely related to gut microbial activity.

The occurrence of depression is related to low serum BDNF levels in the hippocampus and prefrontal cortex, and its pathophysiological mechanism is mainly the damage of neuroplasticity (Zheng et al., 2017). BDNF regulates the transcription and transport of messenger mRNA and is related to the pathogenesis of various neuropsychiatric diseases; however, studies involving neuropsychiatric SLE (NPSLE) have been controversial (Alessi et al., 2022). Prior proof from postmortem investigations showed that people with depressive symptoms had significantly lower BDNF levels in their brains than HC (Martinowich et al., 2007). According to a reported review, patients with significant depressive disorders had abnormally low serum BDNF levels, which increased after antidepressant treatment (Sen et al., 2008). A recent study found that serum BDNF levels were lower in SLE patients with depressive symptoms than in HC, which raises the possibility that serum BDNF levels could serve as a reliable biomarker of depression risk in SLE patients (Zheng et al., 2017). Inflammatory biomarkers (IL-6 and IL-2) have been linked to depression (Lopresti et al., 2014; Buspavanich et al., 2021). Damage to the blood–brain barrier caused by serum IL-6 may contribute to the etiology of NPSLE (Hirohata and Kikuchi, 2021). In a previous investigation, IL-6 increased noticeably in individuals with active SLE and exacerbated inflammation by encouraging non-Treg CD4 T cells to express CCR4 and CCR6 (He et al., 2021). An early study reported that elevated serum IL-2 levels in patients with major depression compared with controls may be a compensatory response to NK cell activity. (Jozuka et al., 2003). There is evidence that the use of IL-2 in cancer treatment induces an inflammatory response system and triggers depressive symptoms (Maes et al., 2001). Hyperactivation of the hypothalamic–pituitary–adrenal (HPA) axis and continuous increase in glucocorticoid levels in patients with depression inhibited the immune response and increased the serum levels of proinflammatory cytokines IL-6 and IL-2 (Ding and Dai, 2019). The HPA axis, in turn, is negatively affected by increased cytokine levels, which aggravate depressive symptoms through neuro-endocrino-immune imbalance in SLE. Hyperimmunism leads to the overproduction of autoantibodies, deposition of immune complexes, release of inflammatory cytokines, and increase in Th2-type cytokine (IL-6) levels. Elevated levels of inflammatory cytokines and immune dysfunction are observed in SLE patients with depression. According to O’Malley et al. (2010) and Singh and Aballay (2019), depression and stress are frequently accompanied by alterations in colonic motility, flora displacement, increased intestinal permeability, impaired intestinal barrier, and alterations in SCFAs content, which cause proinflammatory reactions. Depression might be facilitated by the relationship between the immune and neurological systems. The results of investigations on the gut–brain axis in microbiome are transforming our knowledge of the underlying processes causing depression in SLE patients.

To further explore the association between IL-2, IL-6, BDNF, and abundance of gut microbiome, we established correlation matrices using Spearman’s rank correlation analysis. BDNF and IL-6 were positively correlated with decreased levels of bacteria of the *Agathobacter* genus, whereas IL-2 was positively correlated with the abundance of *Bacteroides*, *Blautia*, *Fusicatenibacter*, and *Collinsella* genera. BDNF was renewed in the relationship between these genera and inflammatory factors. Further studies are required to fully understand this topic. This study also found a significant decline in the number of microorganisms that produce butyrate. The genera *Faecalibacterium* and *Roseburia* showed substantial negative relationships with IL-2 and IL-6, whereas the genera *Faecalibacterium* and IL-6 exhibited strong negative correlations; this implies that increased inflammatory activity, which is linked to the disruption and functioning of the gut microbiome, may contribute to depression in SLE patients. In human intestinal epithelial cells, the *Lactobacillus* genus has demonstrated anti-inflammatory capabilities and also increases BDNF expression in the hippocampus (Wallace et al., 2003; Ranuh et al., 2019). An association between *Lactobacillus* and BDNF was observed in this study. However, the autoinflammatory factor BDNF, which is related to dysbiosis of microbiome, did not exactly correspond with those that differed between SLE-d patients and HC, and specific disorders of the gut microbiome interact with *in vivo* inflammatory factors. BDNF changes may affect the emotions and symptoms of SLE-d patients, which requires further research.

Our study has some limitations. First, our research was conducted only in the Shandong Province of China. Multi-regional investigations may also be of interest in future studies. Second, the sample size of our study was small. To further confirm the findings of this study, studies with a larger sample size are warranted. Finally, there were differences in individual eating habits that could not be strictly controlled, and the lack of dietary information may cloud the results of this study.

## Conclusions

Fecal microbial richness in SLE-d patients decreased, as did the ratio of Firmicutes to Bacteroidetes. We identified specific fecal microbes and their metabolites in SLE-d patients. The occurrence of depression in SLE patients may be predicted by decreased BDNF levels and the elevated levels of autoinflammatory factors (including IL-2 and IL-6) linked to depression, as well as their interaction with gut microbiome.

The mechanism of depression in SLE patients is complex. The proinflammatory response related to gut microbiome dysregulation affects not only the gut–brain axis and cognitive function but also the balance of immune tolerance, which is involved in the occurrence and development of SLE and is also an important cause of anxiety and depression in SLE patients. It is expected that in the future, the gut microbiome will be targeted, perhaps by supplementing the necessary probiotics or changing the diet structure to regulate the gut microbiome disorder and improve the function of the gut–brain axis, so as to reduce the disease activity, inflammation, and depression in SLE patients.

## Data availability statement

The datasets presented in this study can be found in online repositories. The names of the repository/repositories and accession number(s) can be found below: NCBI SRA, PRJNA883162.

## Ethics statement

The studies involving human participants were reviewed and approved by Qingdao Municipal Hospital Affiliated to Qingdao University. The patients/participants provided their written informed consent to participate in this study.

## Author contributions

HanY, YW, and QX conceived and designed the study. HanY^1^, HaoY^2^, QX, YW, and LY collected and organized the clinical data. HanY^1^, HaoY^2^, and YC performed the experiments. HanY^1^, HaoY^2^, and QX performed the microbiome and metabolomic analysis. HanY^1^ drafted the manuscript. QX approved the completion of the manuscript. All authors contributed to the article and approved the submitted version.

## Conflict of interest

The authors declare that the research was conducted in the absence of any commercial or financial relationships that could be construed as a potential conflict of interest.

## Publisher’s note

All claims expressed in this article are solely those of the authors and do not necessarily represent those of their affiliated organizations, or those of the publisher, the editors and the reviewers. Any product that may be evaluated in this article, or claim that may be made by its manufacturer, is not guaranteed or endorsed by the publisher.
