# Supplementary figures and images for: Gut microbiome and fecal metabolic alteration in systemic lupus erythematosus patients with depression
Source: Front Cell Infect Microbiol. 2022 Nov 25;12:1040211. doi: 10.3389/fcimb.2022.1040211 (PMC9732533; doi:10.3389/fcimb.2022.1040211)

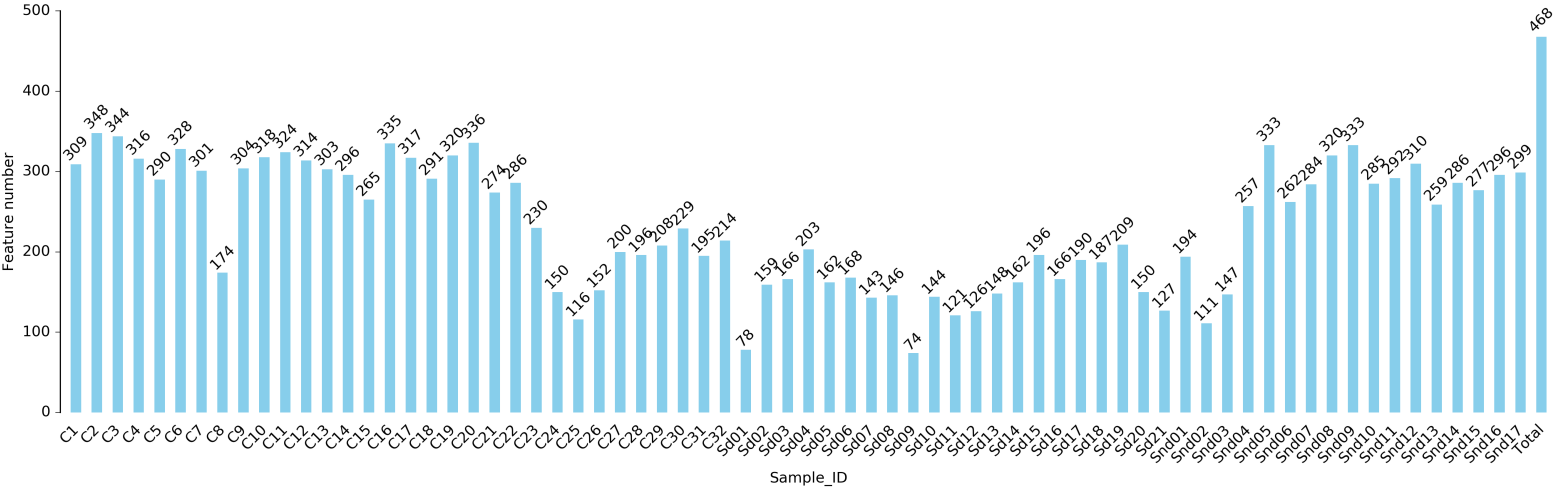

Supplement: Supplementary file 1 [file Image_1.pdf]

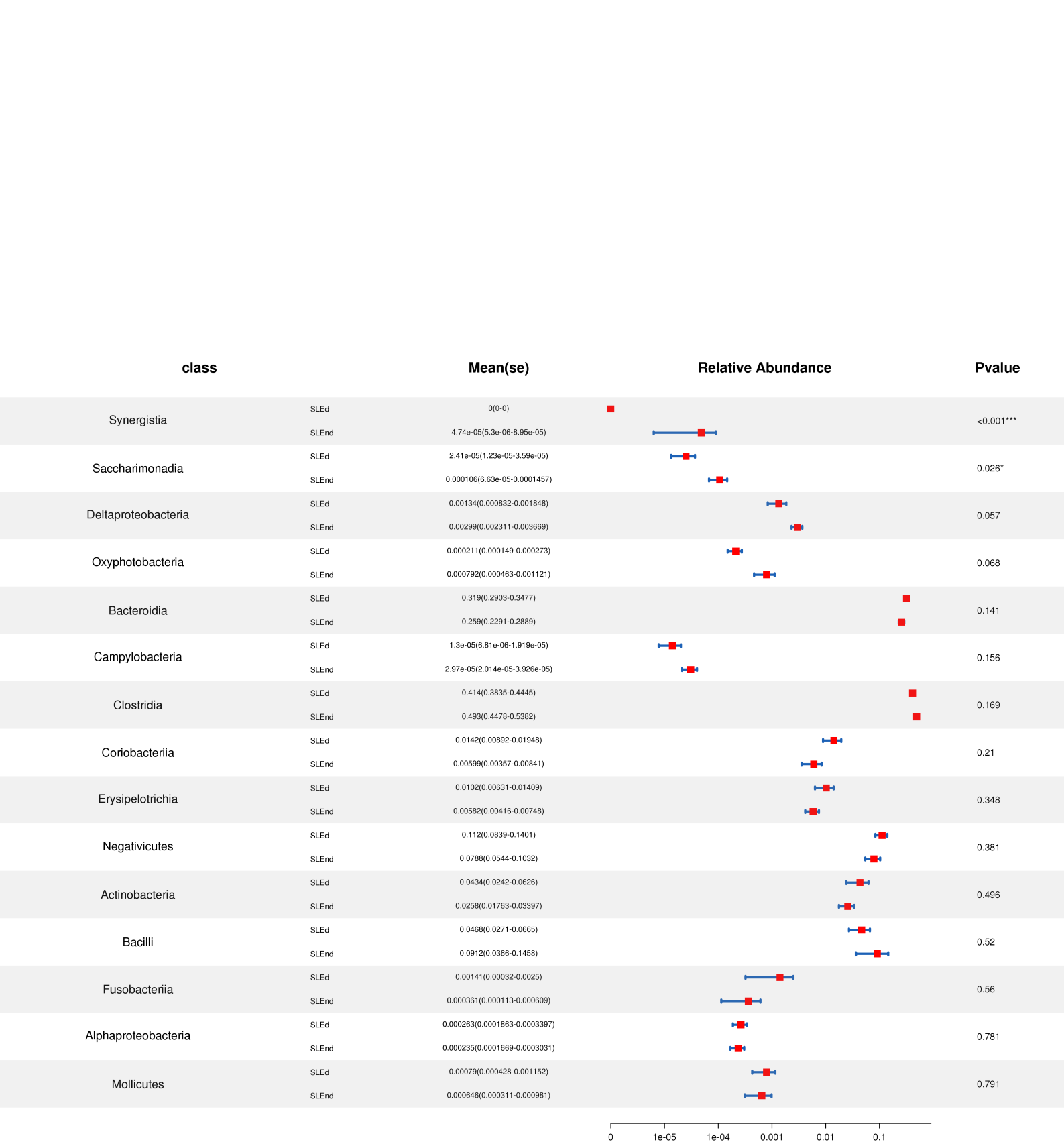

Supplement: Supplementary file 2 [file Image_2.pdf]

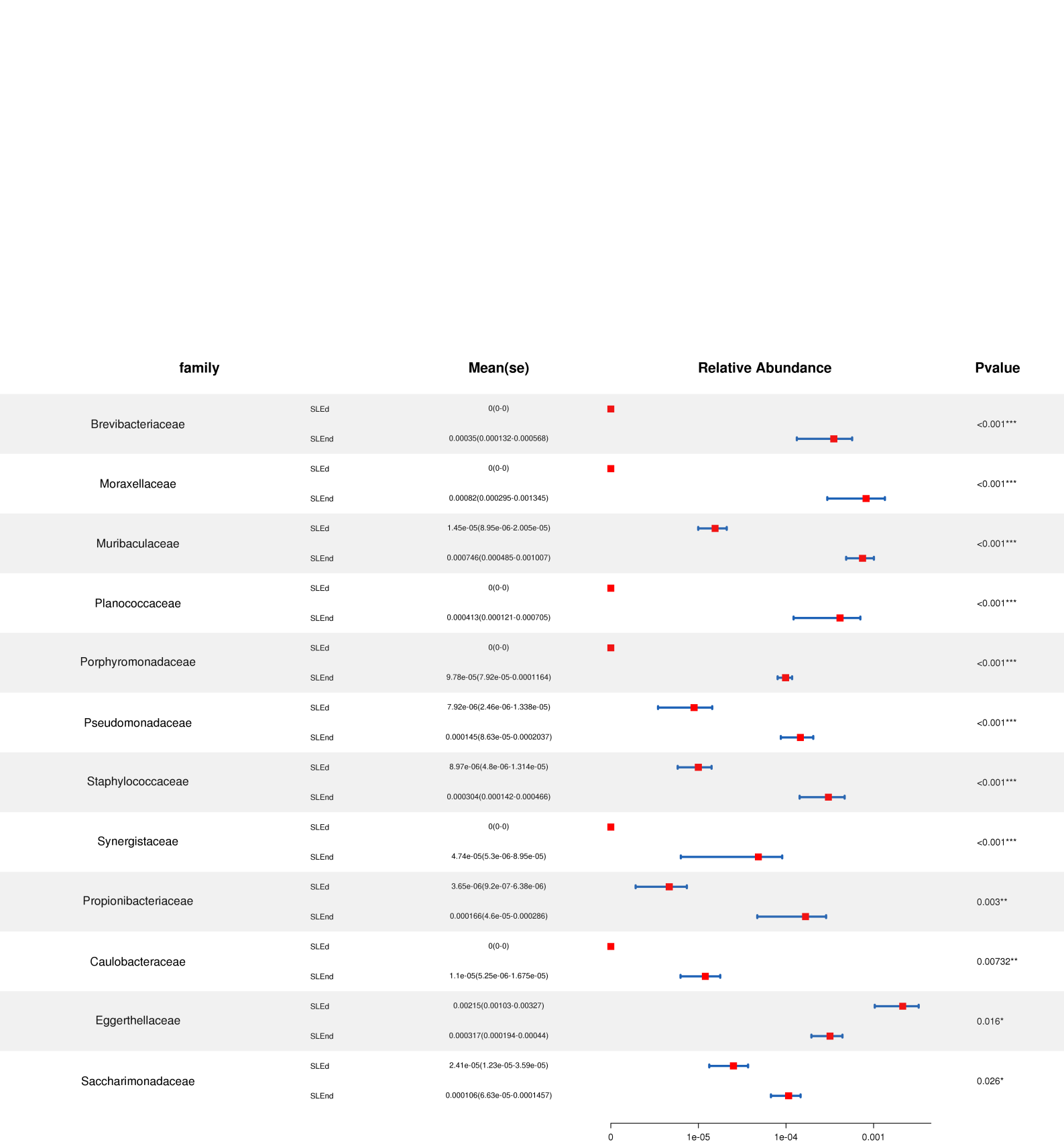

Supplement: Supplementary file 3 [file Image_3.pdf]

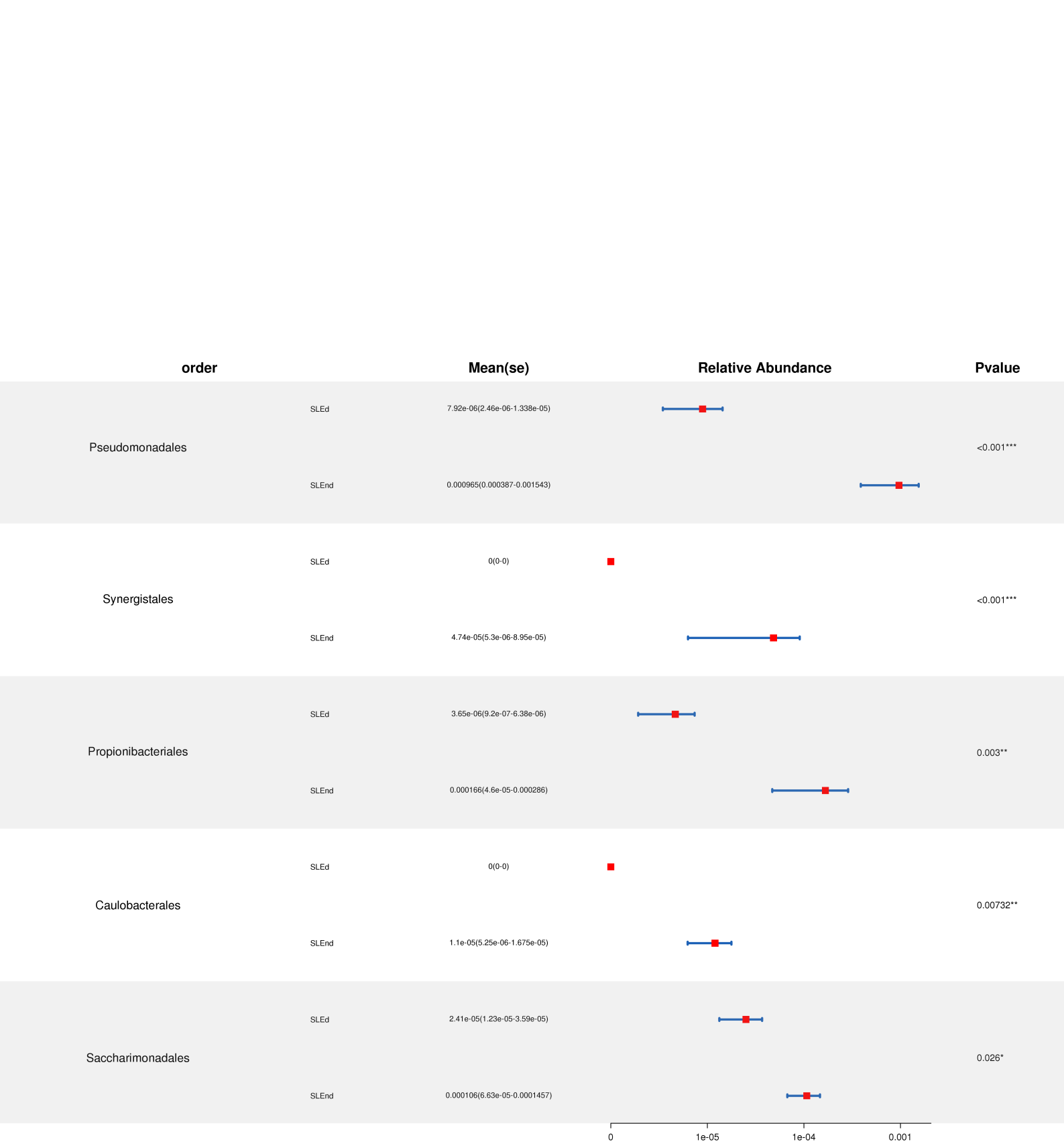

Supplement: Supplementary file 4 [file Image_4.pdf]

# Scores (OPLS-DA)

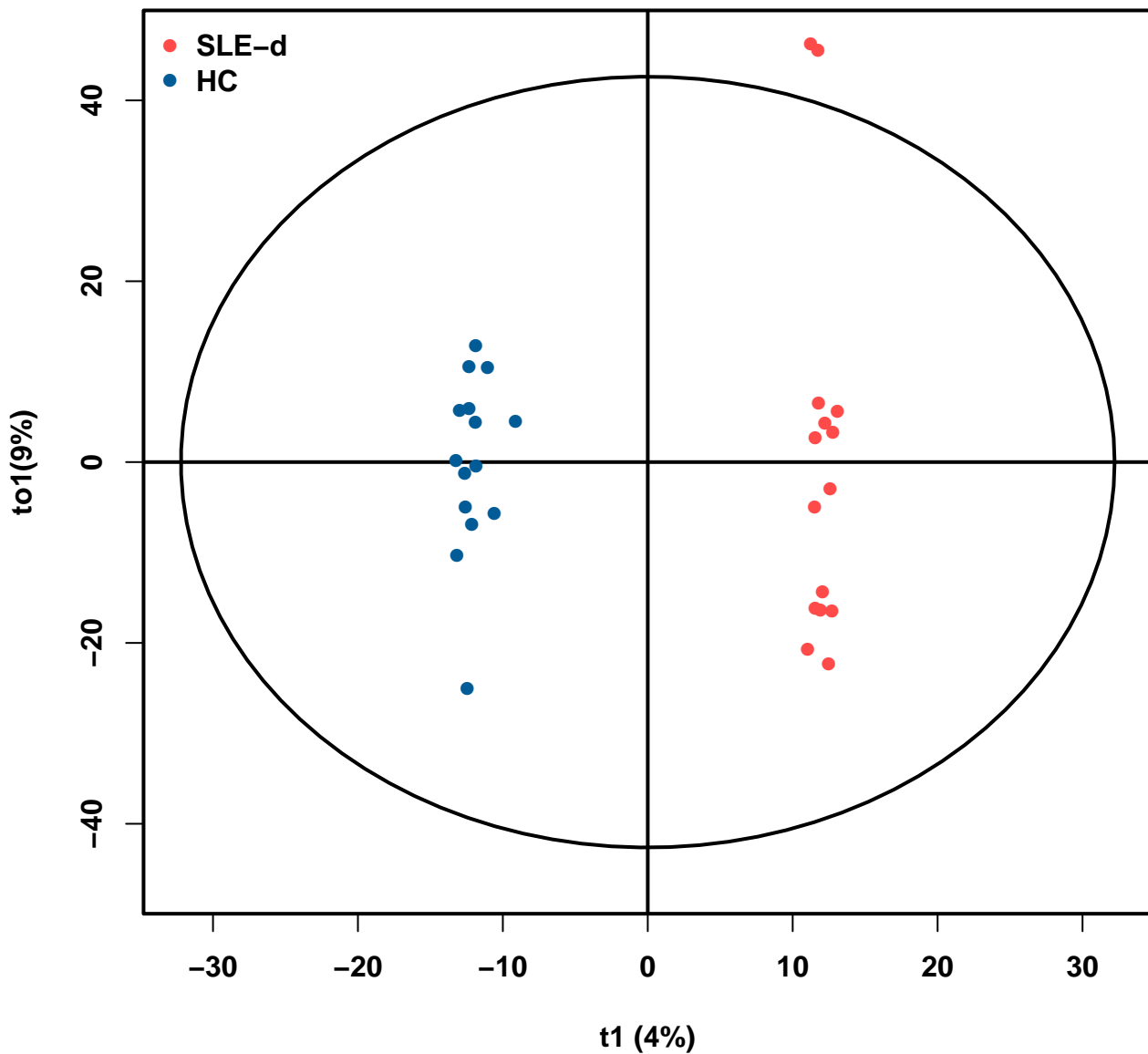

Supplement: Supplementary file 5 [file Image_5.pdf]

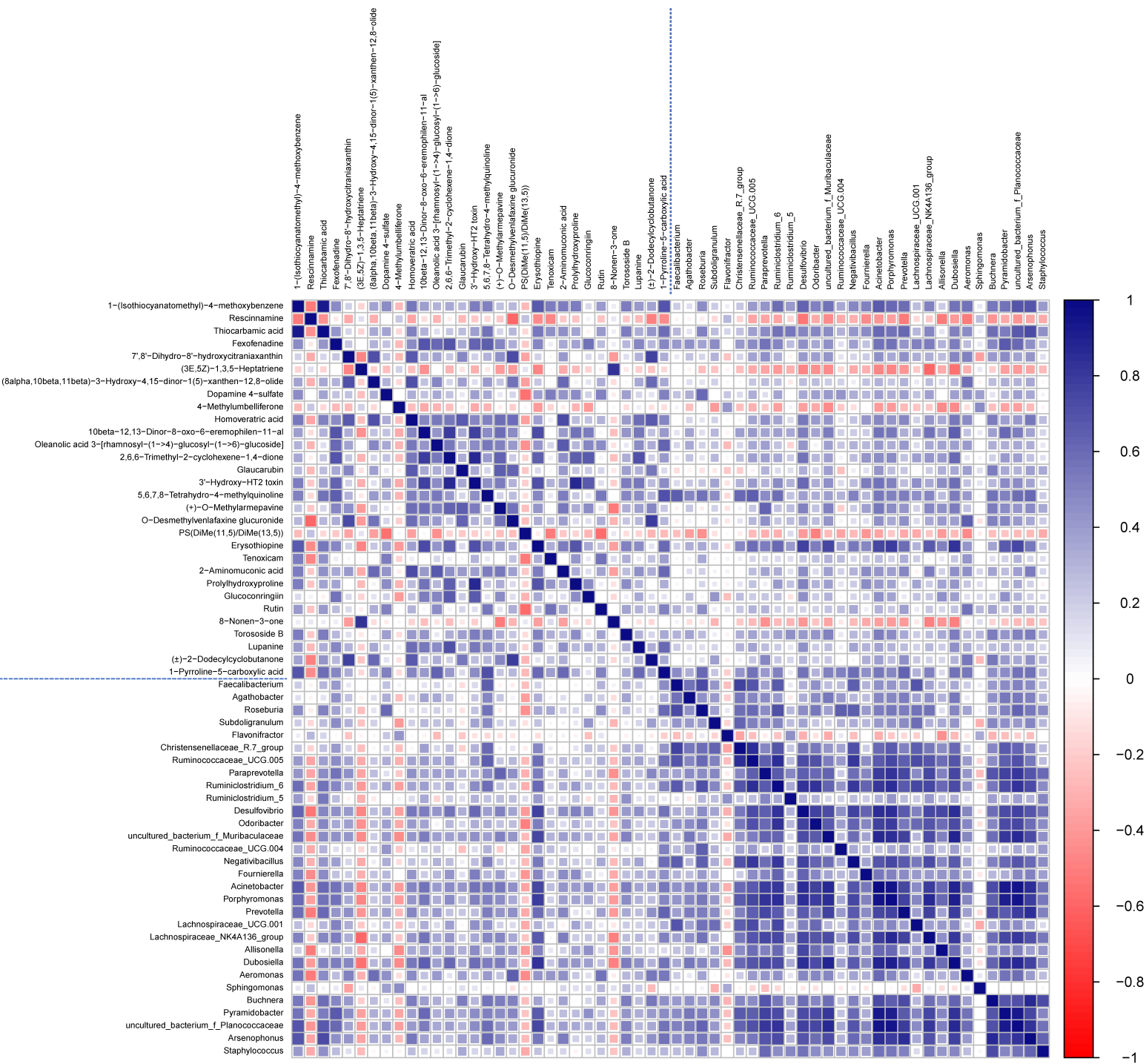

Supplement: Supplementary file 6 [file Image_6.pdf]
